# Improving Vision-Based Motor Rehabilitation Interactive Systems for Users with Disabilities Using Mirror Feedback

**DOI:** 10.1155/2014/964576

**Published:** 2014-09-11

**Authors:** Antoni Jaume-i-Capó, Pau Martínez-Bueso, Biel Moyà-Alcover, Javier Varona

**Affiliations:** ^1^Unitat de Gràfics, Visió i Intelligència Artificial, Department de Ciències Matemàtiques i Informàtica, Universitat de les Illes Balears, E07122 Palma, Spain; ^2^Grup d'Investigació d'Evidència, Estils de Vida i Salut, Department d'Infermeria i Fisioteràpia, Universitat de les Illes Balears, E07122 Palma, Spain

## Abstract

Observation is recommended in motor rehabilitation. For this reason, the aim of this study was to experimentally test the feasibility and benefit of including mirror feedback in vision-based rehabilitation systems: we projected the user on the screen. We conducted a user study by using a previously evaluated system that improved the balance and postural control of adults with cerebral palsy. We used a within-subjects design with the two defined feedback conditions (*mirror* and* no-mirror*) with two different groups of users (8* with disabilities* and 32* without disabilities*) using usability measures (*time-to-start* (*T*_*s*_) and* time-to-complete* (*T*_*c*_)). A two-tailed paired samples *t*-test confirmed that in case of disabilities the mirror feedback facilitated the interaction in vision-based systems for rehabilitation. The measured times were significantly worse in the absence of the user's own visual feedback (*T*_*s*_ = 7.09 (*P* < 0.001) and *T*_*c*_ = 4.48 (*P* < 0.005)). In vision-based interaction systems, the input device is the user's own body; therefore, it makes sense that feedback should be related to the body of the user. In case of disabilities the mirror feedback mechanisms facilitated the interaction in vision-based systems for rehabilitation. Results recommends developers and researchers use this improvement in vision-based motor rehabilitation interactive systems.

## 1. Introduction

Video game console technologies focusing on motion-based inputs, designed to track body motions or body reactions (e.g., *EyeToy*, *Wiimote*, *Kinect*, *Xtion*, and *Move*), are becoming popular and low-cost [1, 2]. These sensors can capture motions of the motor therapy and different studies validated *Kinect* sensor for rehabilitation purposes such as postural control [3], clinical functional analysis and rehabilitation [4], gait retraining [5], activities of daily living rehabilitation [6], and coaching of elderly population [7].

Recent research showed that video games helped to motivate patients in rehabilitation processes [8–10] and rehabilitation results were better with motivated patients [11]. However, different studies concluded that existing commercial motion-based video games were difficult to use in rehabilitation therapy, because they were designed for users with full capabilities [12, 13]. Therefore, researchers developed motion-based video games for motor rehabilitation using the existing commercial motion-based devices: pressure mat based for maintenance of balance in a short-sitting position following spinal cord and head injuries [14]; vision-based for upper limb stroke rehabilitation [15], for chronic stroke recovery [16–18] and to improve the balance and postural control of adults with cerebral palsy [19]; wiimote-based for postural control and functional mobility of cerebral palsy patients [20]; *Kinect*-based to guide and correct therapeutic movements [21], to train static balance [22], and to improve the motor proficiency and quality of life [23]; and haptic-based for stroke rehabilitation [24]. Also, literature reviews about motion-based rehabilitation system were published in the last years [8, 9, 25, 26].

In particular, *Kinect* sensor captures the visual information of the performance of user motions; then it can also consider a vision-based interaction sensor (VBI) [27]. Visual information from the performance of patient actions is a good capture method in motor rehabilitation for two reasons: first, because motor rehabilitation consists of body movements that can be recorded; and second, because VBI is noninvasive and can be used for clients who have difficulties in holding physical devices.

In rehabilitation systems using VBI is critical to provide feedback to users in order to feel in control and help them to understand what is happening [15, 19, 28]. In VBI there is not contact with the interface by means of an interaction device of reference. The user, therefore, always should know when interaction is taking place using visual and audible feedback. For instance, in prior experiences developing vision-based video games for hospitals, medical institutions, and rehabilitation centres [10, 19, 29–31], we detected that users had some difficulties in performing the therapy movements if they could not see themselves in a mirror or on screen.

The advantage of observation and imitation for learning is well studied [32, 33], and mirror movements and imitation learning is recommended in motor rehabilitation [34]. Motor control amends the motion by interaction between visual feedback that recognizes the external space or movement of oneself through vision feedback that refers information about movement and position of body [35]. Moreover, there is evidence that action observation facilitates motor activity [36]. For this reason, mirrors equip motor therapy rooms and they allow the patients to see themselves in order to perform correctly the therapy.

**Figure 1:**
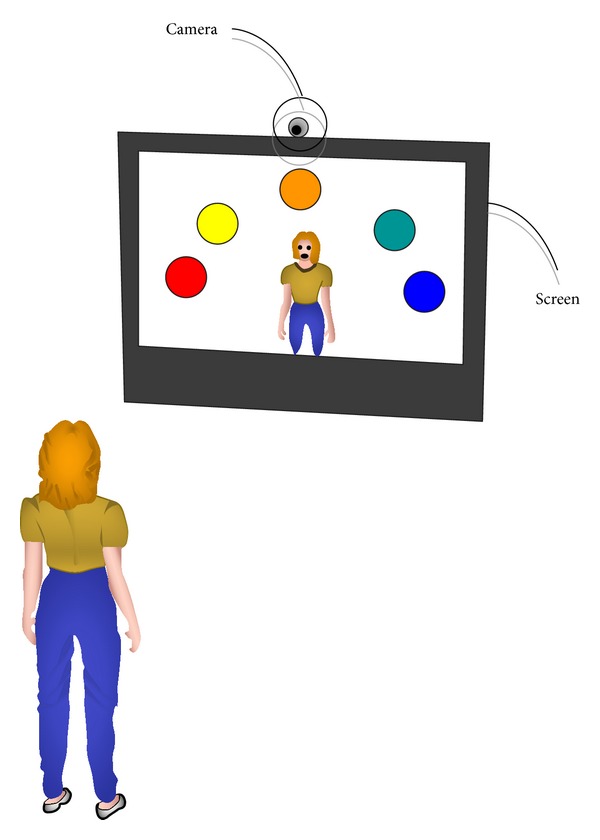
System environment configuration.

In fact, some exiting VBI rehabilitation systems allow the patient seeing themselves on screen, mirror feedback, due to the fact that the users stand in front of a screen and interact with the system using their movements [15, 16, 23]; see Figure 1. It was demonstrated that the user's own image suggested more realism and sense of presence than an avatar figure [37]. The more sense of presence the users have, the more aware of their position and orientation with respect to the interaction elements the users are. Nevertheless, other VBI rehabilitation systems do not implement the mirror feedback [17, 21, 22] because game-based rehabilitation systems designers frequently overemphasize the video game rather than the user interaction. When these games are designed for people with disabilities, the interaction design issues are fundamental to achieve a high patient's motivation. In addition, game interaction design is usually defined without taking into account user's perceptions with regard to their actions in order to achieve the rehabilitation goals.

Different researchers studied the importance and the effectiveness of the augmented feedback in the therapy (information that cannot be elaborated without an external source such as a therapist or a device) [38, 39]. They discovered that visual augmented feedback could improve the performance of the patients on complex motor tasks. However, to our knowledge, there did not exist any study about the importance of the mirror feedback in vision-based rehabilitation systems. Concretely, Sigrist's survey [39] reviewed different types of natural visualization feedback (such as superposition, side-by-side 3D perspective, end-effector movements, and third- and first-person perspective) and only introduced the mirrors in the case of mirror therapy [40].

Our objective was to explore how mirror feedback through interaction could be included into game interaction design in order to observe whether it was possible to improve results in rehabilitation sessions. We performed a user study testing using a game previously designed for balance rehabilitation of cerebral palsy users to explore mirror feedback in vision-based video games. Participants diagnosed with cerebral palsy (CP) with mild to moderate cognitive impairment performed our user study. CP is the most common cause of disabling conditions in children due to the increased survival of low birth-weight infants [41]. The population of adults with CP is growing, as a result of increased longevity, inspiring new research to improve available therapies to achieve better functional abilities. We chose adults with CP for this reason and because many daily activities require both hands and tasks that typically require bimanual coordination [42, 43]. The aim was to experimentally test that the feedback of current games was not enough for understanding the game play by users with cognitive impairment and to demonstrate the feasibility and benefit of including mirror feedback in VBI rehabilitation systems.

The remainder of this paper is organized as follows. The experimental system is presented in Section 2. In Section 3, the experiment is designed and in Section 4 we show the results. Finally, Section 5 discusses the issues observed and the last section is devoted to conclusions and proposed further work.

## 2. Experimental System

The experimental system had been designed to improve the balance and postural control of adults with CP [19]. The system was based on a video game for balance rehabilitation therapy, designed using the prototype development paradigm and features for rehabilitation with video games [15, 28, 44]: feedback, adaptability, motivational elements, and monitoring. We rigorously evaluated the effects of physiotherapy treatment on balance postural control of adult subjects with CP undergoing our experimental system. A 24-week physiotherapy intervention program was conducted with 9 adults from a CP center who exercised weekly in 20-minute sessions. Findings demonstrated a significant increase in balance postural control scores resulting in indicators of greater independence for our participating adults. Scores improved from 16 to 21 points on a scale of 28, according to the Tinetti Scale for risk of falls, moving from* High Fall Risk* to* Moderate Fall Risk*.

**Figure 2:**
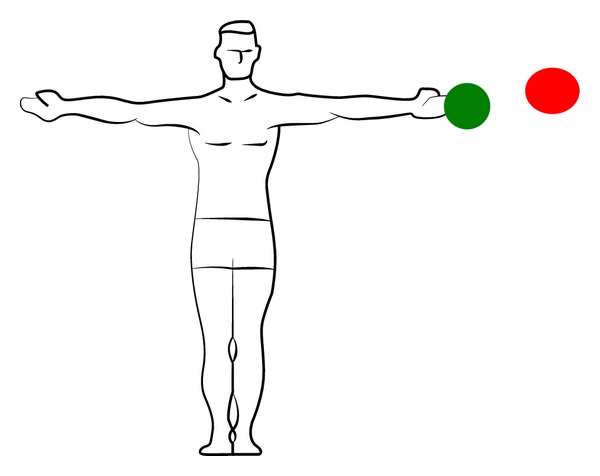
The user is within normal reach of the green object but must change the centre of mass to reach and grab the red object.

We used an active control therapy [45] as interaction method for a video game to improve balance and postural control, increase motivation in clients, and achieve higher adherence to this long-term therapy. The users must interact with objects that cannot be reached without moving the center of mass beyond the base of support (see Figure 2). More specifically, users must remove individual items that appear on the screen by reaching each item with one hand.

**Figure 3:**
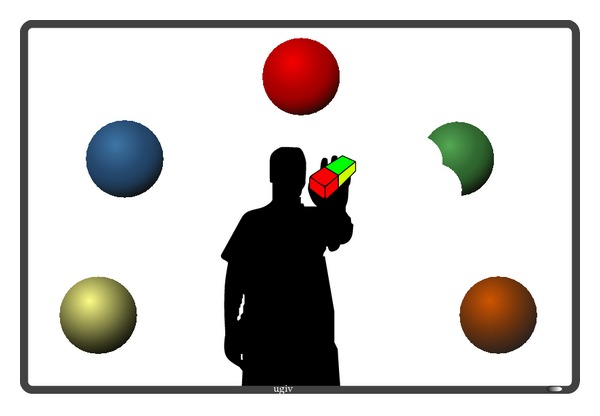
Rehabilitation session introducing mirror feedback.

The video game responds to the actions of the user through different types of feedback, in order for the users to be aware of their current state; see Figure 3.

- A pointer is projected on the user's hand, and the part of the interaction object that intersects with the pointer is erased.
- An auditory feedback is played when an interaction object is completely deleted from the screen.
- Mirror feedback is considered, in such a way that the user can see himself/herself on the screen at all times, so the player's position relative to interaction objects is always known.

Moreover, when the game ends, the user receives different types of visual and auditory feedback, depending on the end game conditions.

The experimental system was developed using C++ programming language, OpenNI as *Kinect* device library, OpenCV as a computer vision library, and Qt as the graphical user interface library.

## 3. Experiment

The experiment performed in this work was aimed at validating that mirror feedback mechanism was important for vision-based rehabilitation systems, especially for the characteristics of the users' set presented.

### 3.1. Participants

Adults diagnosed with CP and with limited voluntary motor control of one or both arms and legs and of the trunk were recruited from the Spanish Association of Cerebral Palsy Centre (ASPACE) in the Balearic Islands. These subjects had mild to moderate cognitive impairment, as shown in Table 1. We used the mini-mental state examination (MMSE) to classify their cognitive impairment because it is a brief and objective screening test and also because it is valid and reliable across a variety of clinical, epidemiological, and community survey studies [46]. The inclusion criteria were as follows:

- age of 20 to 65 years;
- no participation in clinical study published in [19];
- ability to walk with or without technical aids (GMFCS I and II) (Gross Motor Function Classification System);
- ability to understand, learn, and follow simple instructions;
- voluntary agreement to participate in the clinical study.

The exclusion criteria were as follows:

- severe cognitive impairment;
- profound bilateral hearing loss with the use of hearing aids;
- severe visual impairment;
- serious or uncontrolled epilepsy;
- serious or recurring medical complications.

The research team made a request to all adults in the ASPACE. The final study population included 8 adults (7 males), aged 22 to 41 (mean (M) = 33), with CP. Their families signed an informed consent, as legal proxies. Characteristics of the participants are presented in Table 1.

We also included a control group composed of 32 nonpaid volunteers (14 females) aged 19 to 25 (M = 20.4), with no disabilities.

### 3.2. Procedure

In order to explore the importance of mirror feedback in vision-based motor rehabilitation interactive systems, we conducted a user study testing. Specifically, the users tested their own visual representation such as interaction feedback of the video game for rehabilitation.

We were interested in the different users performances in the game interaction enabling or not the explained mirror feedback mechanism; that is, the no-mirror feedback condition was characterized by the absence of such visual feedback. Other feedback mechanisms defined in Section 2 were activated.

We used a within-subjects design with the two previously defined feedback conditions:

MF: mirror feedback (including the user's own visual representation);
NM: no-mirror feedback (absence of such visual feedback).

**Figure 4:**
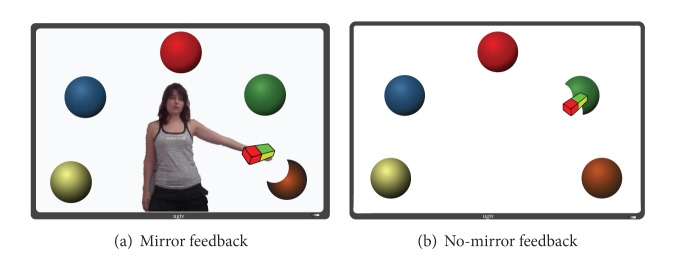
Experimental feedback conditions.

In Figure 4 it is possible to observe the feedback for the two interaction conditions. The user study was divided into two experiments with two different groups of users:

C: control group (users without disabilities);
D: users with disabilities.

For the control group, the user study started with a brief introduction and a demonstration, together with a demographic questionnaire asking about age and previous use of vision-based interaction applications. Participants played two sessions of the designed computer game with the same conditions that the user with disabilities, that is, only moving the upper body part to delete the virtual objects with their hands. For each session the order of conditions (mirror feedback versus no-mirror feedback) was randomly selected so as to balance both interaction conditions across participants.

**Figure 5:**
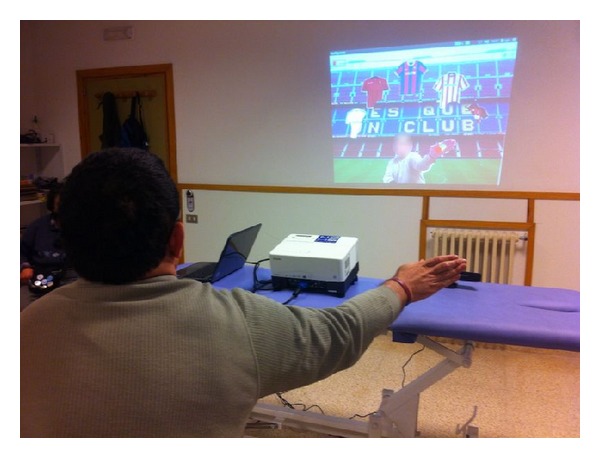
Real performance of the system in ASPACE rehabilitation room using mirror feedback.

For users with disabilities, the game was previously tested on a pilot scheme for a two-month period, attending the rehabilitation center once a week. They practiced the game for at least 20 minutes only with no-mirror feedback condition, and the number of repetitions varied according to participants' tolerance and the physiotherapist's prescription to better manage fatigue. These two months of training were important to ensure a correct understanding of the game and to learn how to carry it out, as well as ensuring a correct parameter adaptation to each user. Once the users correctly understood the game play, participants played two sessions of the designed computer applying the same procedure as for the control group.  Figure 5 shows real performance of the system in ASPACE rehabilitation room using mirror feedback.

### 3.3. Measurements

The gaming was assisted by the physiotherapist and monitored by the research team. All experiments were performed using a PC with this configuration:

- Intel Core2 Duo CPU P8400 @2,26 Ghz;
- 3034 MB RAM;
- Graphic card Mesa DRI mobile Intel GM45 Express;
- Ubuntu 9.10;
- *Microsoft* *Kinect*.

With this configuration the system performance was 30 fps. This result ensured a real-time response from the system [47].

Quantitative measures included logged* time-to-start* (*T*_*s*_) and* time-to-complete* (*T*_*c*_) times. The* time-to-start* measured the time the user interacted with the first virtual object. We interpreted this time as the time taken by the users to orientate their motions with the game interactions. This measure was derived from the observations in the pretest sessions performed with the pilot. In these sessions, users with disabilities had greater difficulty in attaining orientation, and they had trouble knowing their position during play, relative to the interaction objects. This fact was more clearly observable when they had to delete the first virtual object.

The* time-to-complete* measured the time that users needed to complete the deletion of all virtual objects. In the experiment with the group of users with disabilities, the virtual objects were properly located in order to ensure that all the performances achieved the complete deletion goal. Furthermore, in a final questionnaire, the participants selected their preferred interaction feedback for playing the game.

The* time-to-start* is related to effectiveness and* time-to-complete* is related to efficiency of interaction task. According to usability definition [48], it has three aspects: satisfaction, effectiveness, and efficiency. Satisfaction's measures include users' preferences: we had demonstrated that experimental system improved the balance and postural control [19]; that is, the user's objective and the final questionnaire indicated they preferred interaction feedback for playing the game. Effectiveness's measures include quality of solution:* time-to-start* implies first interaction, and users are not able to complete the task if they do not understand the game mechanics and, therefore, start to play. Then, it also has a direct correlation with the task completion and the quality of solution. Efficiency's measures include use of time: tasks completion time (*time-to-complete*).

## 4. Results

Table 2 shows the measured* time-to-start* and* time-to-complete* for users with disabilities using the feedback conditions defined by the experiment. Mirror feedback had better results on the measured times for users with disabilities. Users with moderate cognitive impairment had bigger differences between feedback conditions (mirror versus no-mirror) than users with mild cognitive impairment.

**Figure 6:**
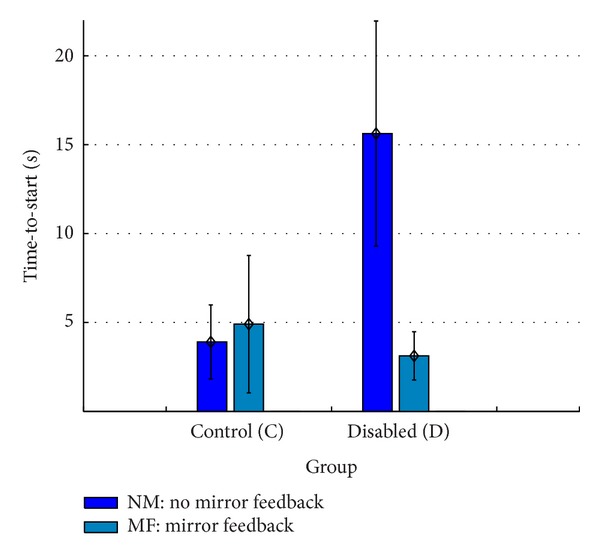
Overview of mean times for the* time-to-start* measure (*T*_*s*_).

**Figure 7:**
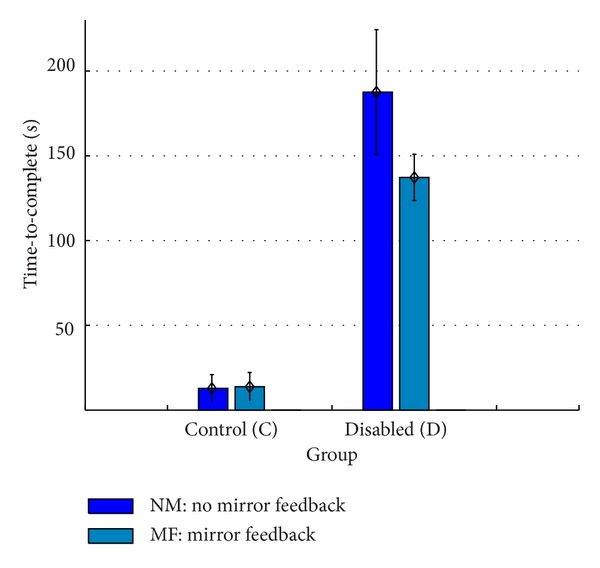
Overview of mean times for the* time-to-complete* measure (*T*_*c*_).

Figures 6 and 7 show mean completed times for both measures, with error bars indicating 95% confidence intervals (CI). They will be further discussed in the following section, based on the interaction feedback.  Table 3 summarizes the influence of mirror feedback in the mean measured times for *T*_*s*_ and *T*_*t*_.

A two-tailed paired samples *t*-test was conducted to evaluate the impact of mirror feedback for each measure in both user groups. Mirror feedback had a highly significant impact on the measured times for users with disabilities (cf.  Table 3). The measured times were significantly worse in the absence of the user's own visual feedback. The control group, on the other hand, completed the experiment with both feedback types with no significant performance differences. However, in the final questionnaire results about interaction feedback preferences, 24 participants of the control group preferred the mirror feedback. For the group with disabilities, seven participants answered that by including mirror feedback mechanisms they gained more control.

Before using the paired *t*-test, we applied a Kolgomorov-Smirnov test of normality (*D* = 0.2587, *P* = 0.1187) and the Wilcoxon signed rank test with continuity correction obtaining a *P* value less than 0.01 (*P* = 0.007015).

## 5. Discussion

Results confirmed our hypothesis that in case of disabilities the mirror feedback mechanisms facilitated the interaction in vision-based systems for rehabilitation. They demonstrated that the implementation of mirror feedback by giving patients the possibility of seeing themselves on screen means that they were conscious at all times of the actions performed relative to the video game. In the user study presented, we proved this claim by means of experiments, showing that a significant improvement of users with disabilities results in the game play. We also observed that users with moderate cognitive impairment had bigger differences between feedback conditions than users with mild cognitive impairment (see Tables 1 and 2).

Different articles reviewed the importance of feedback in motor learning and rehabilitation. To our knowledge, there did not exist any study about the importance of the mirror feedback in vision-based rehabilitation systems. In fact, on one hand, [38] indicated that feedback might enhance motor leaning but there were many areas as yet not examined as the case of mirror feedback. On the other hand, [39] reviewed different types of natural visual feedback such as superposition, side-by-side 3D perspective, end-effector movements, and third- and first-person perspective. However, they did not reference any work about mirrors as natural visual feedback. For this reason, with the aim of seeking deeply articles related to mirror feedback, we also searched at Google Scholar, Web of Science, IEEE Explorer, and ACM Digital Library different combinations of the following key words:* feedback, motor learning, augmented feedback, extrinsic feedback*, and* rehabilitation and mirror*. We did not find any article related to mirrors as natural visual feedback, either.

Finally, we want to discuss the relationship between the mirror feedback and the* game feel* definition used for game design [49]. Game feel is the sensation of the system's response to the player: the kinesthesic qualities of the experience created by coupling with the input device and seeing what happens in the game as a result. In our experience, the input device is the user's own body; therefore, it makes sense that feedback should be related to it as well. In this sense, it is interesting to point out that the results obtained for the control group users (users without disabilities) could be interpreted to mean the mirror feedback mechanism is not significant. However, it should be taken into account that the video game was specifically designed for users with disabilities and its game play was too easy for users without disabilities. It may be interesting to perform another user study with more complex games based on vision-based interaction, in order to properly explore if the introduction of mirror feedback can improve the user experience of vision-based interaction.

Another limitation of our study was the sample size of users with disabilities (8 subjects). However, a post hoc power analysis indicated that with 8 subjects there would be a 90% chance (for *α* = 0.05) that the statistics would have detected a difference greater than 6.6 points in the* time-to-start* measure (we obtained a mean difference of 12.5) and greater than 50 points in the* time-to-complete* measure (we obtained a mean difference of 50.38).

## 6. Conclusion

In this work, we described a feedback implementation that improved results in rehabilitation sessions for users with disabilities, for this reason we recommend developers and researchers use this improvement in vision-based motor rehabilitation interactive systems. We experimented mirror feedback by means of a user study; we considered that it was an appropriate scenario to explore mirror feedback in video games. This claim was supported by the fact that the user's psychological conditions, with mild to moderate cognitive impairment, could mean that the normal feedback of current games was not enough for understanding the game play. In this case, therapy objectives lost effectiveness.

This was particularly interesting in rehabilitation games because the advantage of observation and imitation for learning was known and also because mirror movements and imitation learning was recommended in motor rehabilitation. For this reason, mirrors equip motor therapy rooms and they allow the patients to see themselves in order to perform correctly the therapy.

We observed that users with cognitive impairment had bigger differences between feedback conditions. The higher cognitive impairment user had, the more important feedback was in order to perform correctly the therapy. Results confirmed our hypothesis that in case of disabilities the mirror feedback mechanisms facilitated the interaction in the vision-based systems for rehabilitation. Once the positive effects of mirror feedback mechanism in rehabilitation system were demonstrated, it may potentially be extended to individuals with disabilities, in order to help achieving better functional abilities.

## Figures and Tables

**Table 1:** Characteristics of participants.

User	Age	Physical diagnosis	MMSE
1	22	Cerebral palsy	Moderate
		spastic tetraparesis	cognitive impairment
2	27	Cerebral palsy	Mild
		spastic tetraparesis	cognitive impairment
3	32	Cerebral palsy	Moderate
		spastic tetraparesis	cognitive impairment
4	32	Cerebral palsy	Mild
		mixed spastic tetraparesis	cognitive impairment
5	34	Cerebral palsy	Mild
		spastic tetraparesis	cognitive impairment
6	37	Head trauma	Mild
		spastic tetraparesis	cognitive impairment
7	39	Cerebral palsy	Moderate
		mixed spastic tetraparesis	cognitive impairment
8	41	Cerebral palsy	Mild
		ataxic tetraparesis	cognitive impairment

**Table 2:** Measured *time-to-start* (*T*_*s*_) and *time-to-complete* (*T*_*c*_) for users with disabilities. Mirror feedback (MF) and no-mirror feedback (NM).

User	*T*_*s*_	*T*_*s*_	*T*_*c*_	*T*_*c*_
	MF	NM	MF	NM
1	5	23	150	245
2	2	11	129	160
3	5	26	132	226
4	2	10	126	154
5	2	10	160	174
6	3	15	132	176
7	4	19	148	218
8	2	11	121	148

**Table 3:** Overview of the influence of mirror feedback as interaction feedback for each user group on the mean of the defined time measures.

	*Time-to-start*, *T*_*s*_	*Time-to-complete*, *T*_*c*_
Control group (**C**)	*t*(31) = −1.74, *P* = 0.09	*t*(31) = −0.87, *P* = 0.389
Users with disabilities (**D**)	*t*(7) = 7.09, P < 0.001	*t*(7) = 4.48, P < 0.005

Significant results are printed in bold.
